# A Theoretical Perspective on Molecular Polaritonics

**DOI:** 10.1021/acsphotonics.2c00048

**Published:** 2022-06-03

**Authors:** Mónica Sánchez-Barquilla, Antonio I. Fernández-Domínguez, Johannes Feist, Francisco J. García-Vidal

**Affiliations:** † Departamento de Física Teórica de la Materia Condensada and Condensed Matter Physics Center (IFIMAC), Universidad Autónoma de Madrid, E-28049 Madrid, Spain; ‡ Institute of High Performance Computing, Agency for Science, Technology, and Research (A*STAR), Connexis, Singapore, 138632 Singapore

## Abstract

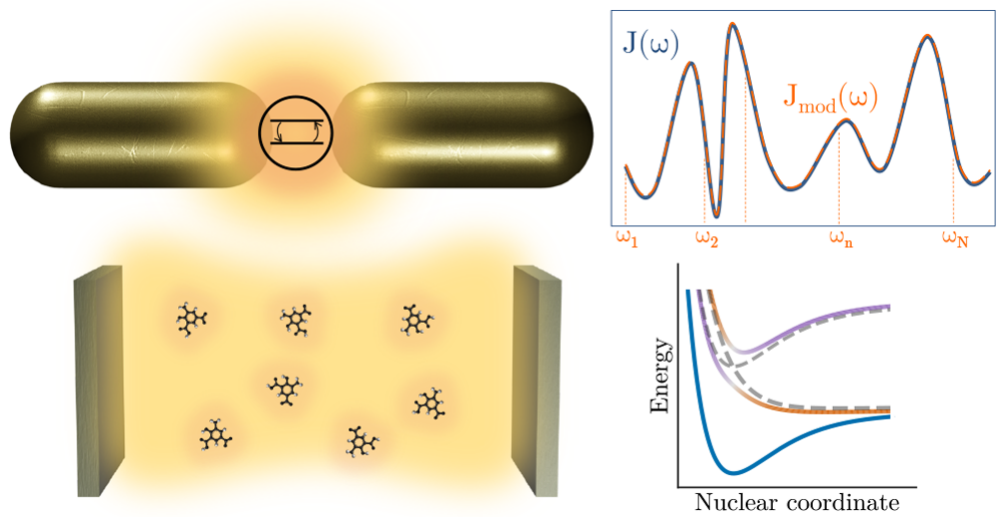

In the past decade, much theoretical research has focused on studying the strong coupling between organic molecules (or quantum emitters, in general) and light modes. The description and prediction of polaritonic phenomena emerging in this light–matter interaction regime have proven to be difficult tasks. The challenge originates from the enormous number of degrees of freedom that need to be taken into account, both in the organic molecules and in their photonic environment. On one hand, the accurate treatment of the vibrational spectrum of the former is key, and simplified quantum models are not valid in many cases. On the other hand, most photonic setups have complex geometric and material characteristics, with the result that photon fields corresponding to more than just a single electromagnetic mode contribute to the light–matter interaction in these platforms. Moreover, loss and dissipation, in the form of absorption or radiation, must also be included in the theoretical description of polaritons. Here, we review and offer our own perspective on some of the work recently done in the modeling of interacting molecular and optical states with increasing complexity.

## 1 Introduction

Polariton is a general term used to describe a hybrid light–matter excitation and has been employed in many different situations in the history of physics.^1^ In this Perspective, we focus on a small subset of these situations, namely, those in which electronic transitions in molecules (often organic dyes) are used to provide the material component. Even within this small subset, a wide range of new phenomena are enabled by polariton formation, among them Bose–Einstein condensation and polariton lasing,^2,3^ quantum information processing,^4^ long-range excitation transport,^5,6^ and control of chemical reaction rates.^7^ After a short overview of basic physical concepts, we discuss our view on the current state of the field and the challenges it faces, interesting recent developments, and promising future directions. Despite the focus on molecules, we here restrict the discussion mostly to “physical” properties and ignore “chemical” properties such as (photo)reactivity that have been the focus of a recent related perspective on polaritonic chemistry.^8^

**Figure 1:**
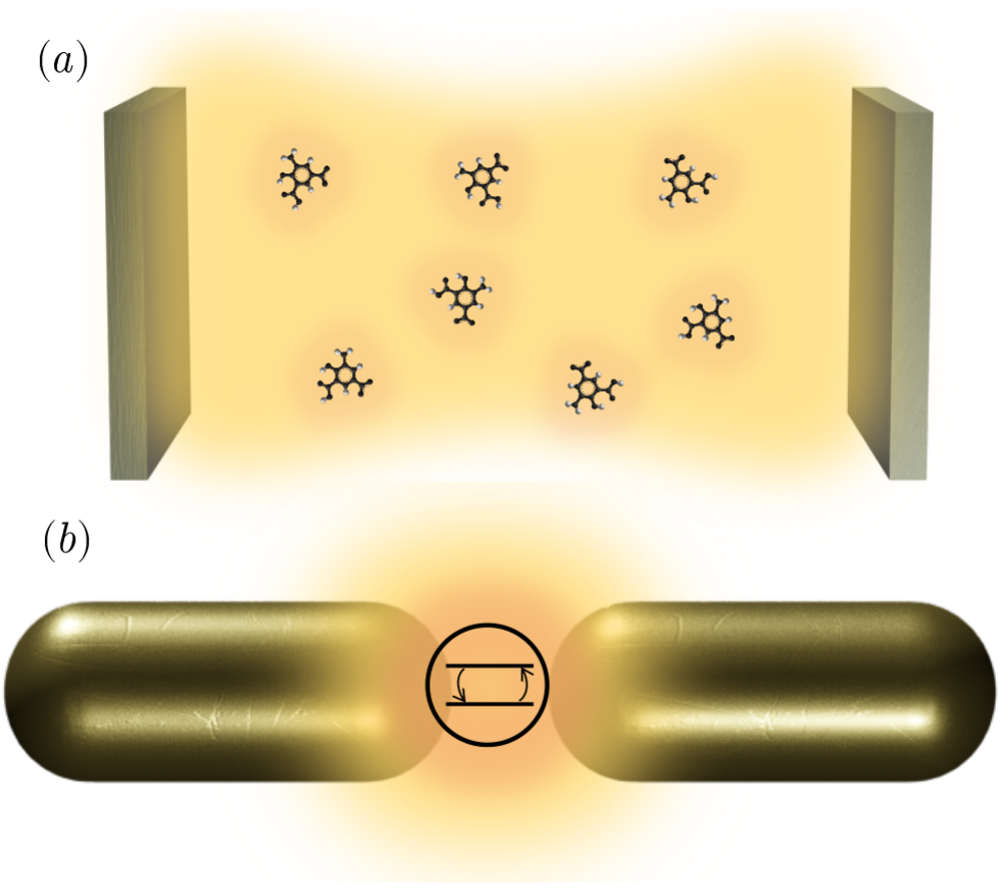
Schematic representations of typical situations in molecular polaritonics. (a) Molecular ensemble inside a photonic microcavity and (b) single emitter coupled to a plasmonic nanoantenna.

Polaritons arise when the interaction strength between light modes and material excitations in a system becomes large enough that the system enters the strong coupling (SC) regime.^9,10^ In this regime, the eigenstates of the system are not even approximately represented by pure material or pure electromagnetic excitations (photons). The resultant hybrid light–matter excitations are then called polaritons. They were first observed in 1965^11^ in a crystal, while surface exciton–polaritons where observed approximately a decade later.^12^ Before introducing strong coupling in more detail, we note that the apparently simple question of what should be called a “light mode” is actually somewhat subtle, and, as for any question of semantics, its answer is to some degree arbitrary. The only “pure” light modes are free-space modes in vacuum. When using any material structure that confines the EM field (often called “cavity” in general in this context), the resulting “cavity photon modes” are always partially material excitations.^8,13^ As an operational definition, the cavity modes are usually understood as the modes supported by those parts of the full system for which the relevant dynamics can be well-approximated through macroscopic electromagnetism under linear response. This distinction leads naturally to the framework of macroscopic QED,^13−21^ discussed in more detail below. The resulting electromagnetism (EM) modes then include those of optical (Fabry–Pérot) cavities^22,23^ (as depicted in Figure 1a), photonic crystals,^24,25^ and often also plasmonic nanostructures (as illustrated in Figure 1b), whose resonances are formed due to geometrical restriction of the free electron motion in metals and allow strongly subwavelength field confinement to be achieved.^26−28^ However, plasmon modes are physically quite distinct from “optical” modes. In Fabry–Pérot or photonic crystal cavities, the energy is mostly stored in the electric and magnetic fields, and the dielectric functions of the materials can often be treated as approximately constant within the relevant range of frequencies. In contrast, the energy in plasmonic resonances is stored in the electric field and the kinetic energy of the electrons,^29^ such that the resulting modes are more correctly referred to as surface plasmon polaritons themselves. We note that such structures should more correctly be called plasmonic “resonators” or “antennas” rather than “cavities”, although the latter use has become common in the field.

Plasmonic and other deep-subwavelength cavities are also somewhat special in another regard. The EM fields at close distance to such structures, where emitters are typically placed, are dominantly electrostatic (or more precisely, quasistatic), as they are due to the Coulomb fields of the displaced charges (e.g., electrons oscillating collectively). This has fundamental consequences on the light–matter interaction, as the local electric fields are then to a good approximation purely longitudinal (corresponding to Coulomb interactions). In the standard Coulomb gauge, they arise from the scalar potential and not the vector potential that describes propagating radiation modes (i.e., transverse EM fields).^8,13,30^ As an additional complication, plasmonic systems can have complex internal dynamics after excitation, e.g., leading to hot-electron generation, which in turn can have significant effects on chemistry.^31,32^ In this Perspective, we do not discuss such hot-electron effects.

Once a suitable definition of what constitutes the light modes in the system has been chosen, it becomes possible to distinguish between the weak and strong-coupling regimes within that model. We note that strong coupling itself is not necessarily a quantum effect and can often be modeled through classical electromagnetism.^10,33^ In that case, the material excitations are represented through the dielectric function of the medium (e.g., through a resonance of Drude–Lorentz form) or through a polarizable dipole for single emitters.^34,35^

For a single quantum emitter approximated as a two-level system coupled to a single photon mode within the rotating wave approximation, the dynamics is described by the Jaynes–Cummings model.^36^
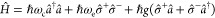
1where *â* and *â*^†^ are the annihilation and creation operators of the photonic mode inside the cavity, σ̂^±^ are the quantum emitter excitation and de-excitation operators given by the corresponding Pauli matrices, *g* is the coupling strength between both constituents, and ω_c_ and ω_e_ are the cavity mode and emitter frequencies, respectively. When the emitter and photon mode are on resonance, the strong-coupling regime is entered when their mutual interaction overcomes decoherence in the system, i.e., *g* ≳ γ, where γ is a typical decoherence rate (the exact criterion that should be used is somewhat arbitrary with several valid choices; see ref (10) for a discussion). In this regime, energy exchange between light and matter becomes a coherent process: if only one of the components is initially excited, this energy exchange is seen as an oscillatory behavior of the population between both subsystems, the so-called vacuum Rabi oscillations occurring at the vacuum Rabi frequency (or Rabi splitting), which in general is given by
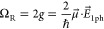
2where μ⃗ is the transition dipole moment of the emitter and  is the quantized electric field strength of the mode, associated with one photon, at the emitter position. Here, *V* is the effective volume of the cavity mode, which gives an estimate of the volume within which a photon is confined in such a structure, the correct definition of which in nanophotonic devices has been the subject of intense theoretical activity over the past decade.^37−39^ Furthermore, we note that its definition in systems with translation symmetry (e.g., a 2D Fabry–Pérot cavity consisting of formally infinitely extended parallel mirrors) is somewhat arbitrary, as it depends on the chosen quantization volume and carries no physical information in that case.

**Figure 2:**
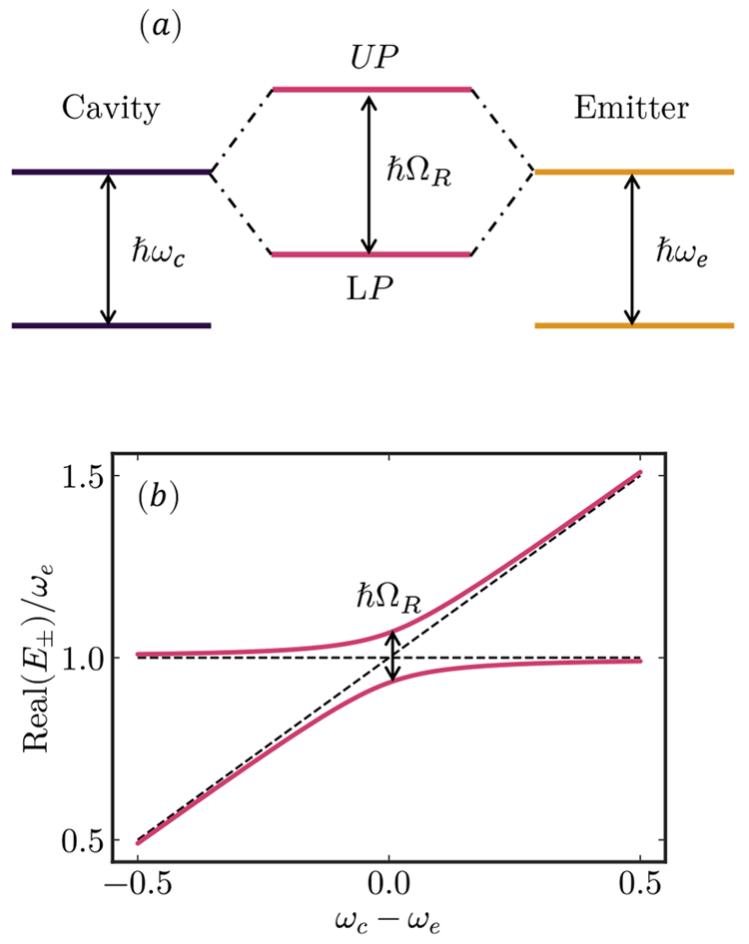
(a) Scheme of the hybridization of one EM mode (purple) and one quantum emitter (yellow). In the strong-coupling regime, an upper (UP) and lower (LP) polariton are formed and are separated by the Rabi splitting Ω_R_. (b) Energies of the polaritons under nonzero detuning. The dashed lines show the uncoupled frequencies.

Physically, vacuum Rabi oscillations correspond to the situation where a photon can be emitted and reabsorbed several times before it disappears from the system. In these conditions, the eigenstates of the coupled system are hybrid light–matter states, and a thorough understanding of the system can only be reached by considering the coupled system as a whole. The two original excited states (emitter and photon) transform into polaritonic states that are shifted up and down in frequency by the coupling strength, with their difference in energy given by the Rabi splitting. These two states are conventionally called the lower polariton (LP) and upper polariton (UP). Figure 2a schematically shows the formation of the upper and lower polaritons when the cavity mode and the emitter frequency are on resonance, while Figure 2b shows the evolution of the polariton frequencies when ω_c_ and ω_e_ are detuned from each other.

We mention here that some care should be taken to distinguish between the concepts of polaritonic states and polaritons. The former are the hybrid eigenstates of the coupled system, which do not depend on the state of the system at any point in time and in that sense always exist. The latter are the excitations of the system in a quasi-particle picture, and thus only exist when the system is in one of the polaritonic states. For one or a few emitters, each polaritonic state is unique. However, for macroscopic systems containing many emitters, excitons behave (approximately) as bosons,^40^ a feature that is inherited by the polaritons. The excitons and photonic modes are then approximately harmonic oscillators, and their coupling produces new (polaritonic) normal modes. Each of these *modes* contains a (formally) infinite number of polaritonic Fock *states* |0⟩_*P*_, |1⟩_*P*_, |2⟩_*P*_, .... In this picture, having *n* polaritons in mode *P* means that the system is in state |*n*⟩_*P*_. In the literature, the distinction between polaritons, polaritonic states, and polaritonic modes is not always made explicit, which can cause some confusion. Which of the three is meant is usually clear from the context.

For single emitters, reaching the SC regime is extremely challenging, as the single-emitter coupling strength *g* has to become comparable to the emitter and cavity decoherence rates. This can be achieved by either increasing the coupling strength or decreasing the decoherence rates sufficiently. This was first achieved in 1985 by working with long-lived emitters and cavities at cryogenic temperatures, in a microwave cavity with superconducting mirrors.^41^ Rabi oscillations in the system were explicitly measured two years later.^42^ In 2004, the SC regime for a single quantum dot in a semiconductor micropillar cavity was achieved, with a Rabi splitting of ∼100 μeV.^43^ These approaches, where the absolute coupling strength is a small fraction of the excitation energy, necessarily require very long-lived emitters and cavity modes, which in turn implies cryogenic temperatures. More recently, the strong-coupling regime has been approached at room temperature for single quantum emitters by using extremely localized surface plasmons in narrow gaps,^44,45^ which support light confinement in deeply subwavelength volumes.^46−48^ The estimated single-emitter Rabi splitting achieved in refs (44) and (45) was 120 and 90 meV, respectively, both at the limit of the strong-coupling regime. Very recently, strong coupling and quantum nonlinearity have been observed for a single molecule at cryogenic temperature,^49^ where the molecule behaves as an effective two-level system.

Strong light–matter coupling is much easier to achieve in the collective case where an ensemble of *N* close to identical quantum emitters interacts with a photonic mode (as described by the Tavis–Cummings model^50^). In that case, the effective coupling strength increases with the number of emitters as *g*_*N*_ = *g*√*N*.^40^ This enhancement significantly simplifies entering the strong-coupling regime and is the basis for most experiments in the field of molecular polaritonics. It occurs because an excitation in this case can be coherently distributed over the *N* emitters, forming a so-called bright state with increased light–matter coupling. At the same time, all *N* – 1 orthogonal ways of distributing an excitation over the emitters show negligible coupling to the cavity mode due to destructive interference between the dipole transitions in the different emitters. These superpositions are the so-called dark states, which play a major role in molecular polariton dynamics.^51,52^

Collective strong coupling was first realized in 1975 using molecular vibrations coupled to surface phonon polariton modes^53^ and soon after for molecular excitons coupled to surface plasmon polaritons^54^ and atoms coupled to high-*Q* cavities, first in the microwave^55^ and then in the optical^56^ regime. Strong coupling to semiconductor (Wannier) excitons was first realized in 1992.^57^ Such systems reach Rabi splittings in the range of 1–20 meV.^57−59^ Organic semiconductors support much larger Rabi splittings, Ω_R_ ≳ 100 meV, due to their high density and large dipole moments, so that strong coupling can be observed at room temperature.^60^ We note that the maximally reachable Rabi splitting for a given material is determined by the density of dipoles, but largely independent of the specifics of the photon mode.^61−63^ This can be understood by noticing that
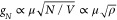
3where ρ is the molecular number density, and we have used the fact that the effective mode volume is related to the physical volume occupied by the mode. The number of molecules interacting with the mode is thus proportional to the molecular density times the volume. A more careful calculation shows that the Rabi splitting depends on the dipole density multiplied by a “filling factor” between 0 and 1 that determines what fraction of the mode volume is filled with the molecular material (weighted with the position-dependent quantized field strength).^39,61^ When a cavity is completely filled with the material in question, the Rabi splitting is equal to the bulk polariton splitting obtained by Hopfield in 1958.^64^ These facts explain why similar Rabi splittings have been observed in the literature for very different photonic systems, such as Fabry–Pérot cavities,^60^ plasmonic surfaces,^65^ plasmonic hole arrays,^66^ isolated particles and arrays of them,^67,68^ and nanoparticle-on-mirror setups.^45^ Several kinds of organic materials can reach the so-called ultrastrong-coupling regime,^69^ in which the Rabi splitting is a significant fraction of the bare excitation energy, with record values close to and above Ω_R_ = 1 eV.^70−72^ Apart from the molecular density, the alignment of the dipole moments of the molecules relative to the electric field inside the cavity also modifies the Rabi splitting. While most experiments use disordered materials with randomly oriented molecules, a factor of up to √3 can be gained in the Rabi splitting when the molecules are perfectly aligned with the electric field. Exploiting this has allowed large Rabi splittings up to Ω_R_ = 1.8 eV to be achieved.^73^ The large reachable coupling strengths in organic materials also mean that cavity modes with very large decay rates κ (or equivalently, short lifetimes τ = 1/κ or low quality factors ω_c_/κ) can be used while still reaching the strong-coupling regime.

As noted before, organic molecules are very well-suited for reaching large Rabi splittings due to the large transition dipole moments and high densities. However, they have complex internal structure due to their rovibrational degrees of freedom and often cannot be approximated as two-level systems. On one hand, this complicates their use and study as idealized (two-level) quantum emitters. On the positive side, this opens up the opportunity to modify their internal structure and dynamics through strong light–matter coupling or conversely to exploit the internal dynamics to achieve new photonic functionalities. The former type of applications are exemplified by the field of polaritonic chemistry, which aims at modifying chemical processes such as photochemical reactions through strong light–matter coupling.^8^ The latter type of applications typically rely on the fact that molecules show strong exciton–vibration interactions, such that molecular vibrations can drive polariton relaxation or transfer between different polaritonic states.^74,75^ This can enable processes such as organic exciton–polariton lasing and condensation^3,76,77^ or energy transfer between different molecular species even over long spatial distances.^5,6,78−82^ Note that it has been also shown that, even under the two-level system approximation, SC phenomena involving organic molecules offer possibilities for nonclassical light generation not attainable by means of other types of quantum emitters.^83,84^

When describing light–matter interactions in molecular systems, in particular in the strong-coupling regime, including all the degrees of freedom in both constituents is an arduous task. Then, many models are focused on taking into account the complexity of one of them; that is, the theoretical effort is focused either on the description of the complexity of the photonic structures or to include to some extent the vibrational structure of the molecules. In what follows, we summarize some of the theoretical challenges that remain in both paths.

## 2 EM Field Quantization in Complex Geometries

Many different kinds of “cavities” can be used to achieve strong coupling in the collective regime, while few- or single-molecule strong coupling necessarily requires deep-subwavelength confinement of light. In general, any cavity setup is determined by “macroscopic” structures consisting of large numbers of atoms, such as Fabry–Pérot cavities, photonic crystals, and metallic nanoparticles or surfaces. Within these setups, one or several microscopic quantum emitters such as atoms, molecules, or point defects are placed. As discussed above, it is then customary to treat the macroscopic structure through Maxwell’s equations and formally treat the modes arising from these equations as the EM modes of the system.

In order to describe light–matter interactions at a quantum level, these EM modes have to be quantized, which is significantly more challenging than the quantization of free EM modes in conventional quantum electrodynamics. For one, the presence of material structures complicates the solution of eigenmodes, which often is only possible numerically. Nowadays, many commercial and open source packages are available to solve Maxwell’s equations in these situations. Furthermore, these light modes will be lossy, often highly so, due to both material losses and leakage to the far field. Only in some approximations do lossless states exist, e.g., when assuming the existence of perfectly conducting mirrors or infinitely long lossless and defect-free waveguides, etc. None of these are typically good approximations for the kinds of structures used in molecular polaritonics. Still, when losses are small enough, it can be a reasonable strategy to quantize fully bound modes in a fictitious lossless system and then treat the losses as small perturbations on top of that.

Alternatively, when dealing with small enough nanoparticles with localized resonances (such as plasmonic nanoparticles) for which radiative losses are small due to inefficient emission, the so-called quasistatic approximation is often applicable. In this approximation, retardation effects, and therefore EM propagation into free space (or bulk dielectric media), is neglected, resulting in purely longitudinal fields. In this limit, semianalytical solutions are often again possible, e.g., using transformation optics,^85−87^ with the resulting modes being fully bound while still describing the material losses. Since subwavelength confinement is a prerequisite for the quasistatic approximation, these material losses will always be significant.^29^ One advantage of the quasistatic approximation is that the EM modes can be described by a scalar potential, which simplifies the treatment of beyond-dipole interactions.^88−90^ Another advantage of the quasistatic approximation is that for metals described by a dielectric function of Drude form the resulting eigenmodes will always correspond to uncoupled Lorentzians in the spectral density (discussed in more detail below), which allows for a straightforward quantization procedure of the resulting modes.^88,90,91^ Radiative losses can also be included a posteriori, e.g., by calculating the effective dipole moment of the localized resonances.^88,92,93^ These quasistatic treatments of plasmonic modes have led to analytical insights into different aspects of strong light–matter coupling in metal nanostructures. On one hand, they have shown that molecular degrees of freedom such as the presence of light-forbidden transitions can be harnessed to tailor polaritonic properties.^88,92^ On the other hand, they have also revealed different strategies to exploit the polaritonic (originally excitonic) quantum nonlinearities for nonclassical light generation in these deeply subwavelength systems.^93^

When the quasistatic approximation is not appropriate and retardation effects have to be taken into account, the most general and powerful approach to nonetheless obtain a quantized description of the EM field is macroscopic QED. This is a formalism that quantizes the EM field in arbitrary structures, including dispersive and absorbing materials. A particularly appealing feature of this approach is that the quantized EM modes are fully described by the dyadic Green’s function **G**(**r**, **s**, ω) of the classical Maxwell equations, which can be obtained from numerical solvers. This can be conceptually understood from the fact that Maxwell’s equations are the wave equations describing the dynamics of EM fields, which remains true after quantization. A recent review about macroscopic QED in the context of nanophotonics can be found in ref (13). We note here that, although the classical description of the EM environment is valid for a wide variety of physical situations, it breaks down when the material and the emitters are physically close enough that electronic wave functions overlap,^94,95^ which happens at subnanometer separations.

Within macroscopic QED,^19,20^ the Hamiltonian for multiple emitters in the multipolar coupling scheme (Power–Zienau–Woolley picture) and within the dipole approximation can be written as
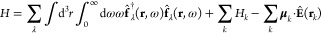
4where **f̂**_λ_(**r**, ω) and **f̂**_λ_^†^(**r**, ω) are bosonic annihilation and creation operators of the medium-assisted EM field, with λ = {e, m} labeling electric and magnetic contributions. *H*_*k*_ and **μ**_*k*_ are the bare Hamiltonian and dipole operator of emitter *k*. The electric field operator **Ê**(**r**) = **Ê**^+^(**r**) + **Ê**^–^(**r**), where **Ê**^+^(**r**) = (**Ê**^–^(**r**))^†^ is defined by
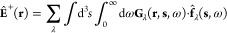
5where **G**_λ_(**r**, **s**, ω) is closely related to the standard dyadic Green’s function **G**(**r**, **s**, ω).^19,20^

It can be shown that the action of the EM environment on the emitters is fully characterized by the generalized spectral density
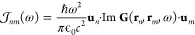
6which is determined by the classical dyadic Green’s function and which we give here directly in the form generalized to several emitters and several possible dipole orientations per emitter.^96^ Here, *n* and *m* are combined labels for the emitter and the transition directions that are taken into account (up to three per emitter), with **u**_*n*_ the unit vector describing the orientation and **r**_*n*_ the position of the corresponding emitter. The diagonal elements *n* = *m* define the coupling between the EM field and each dipole transition, while the off-diagonal elements *n* ≠ *m* define the photon-mediated interaction between emitters. Notice that in the weak-coupling limit, the Markov approximation can be used to obtain decay rates and environment-induced dipole–dipole interactions, which are determined by the imaginary and real parts of the Green’s function, respectively.^97^

**Figure 3:**
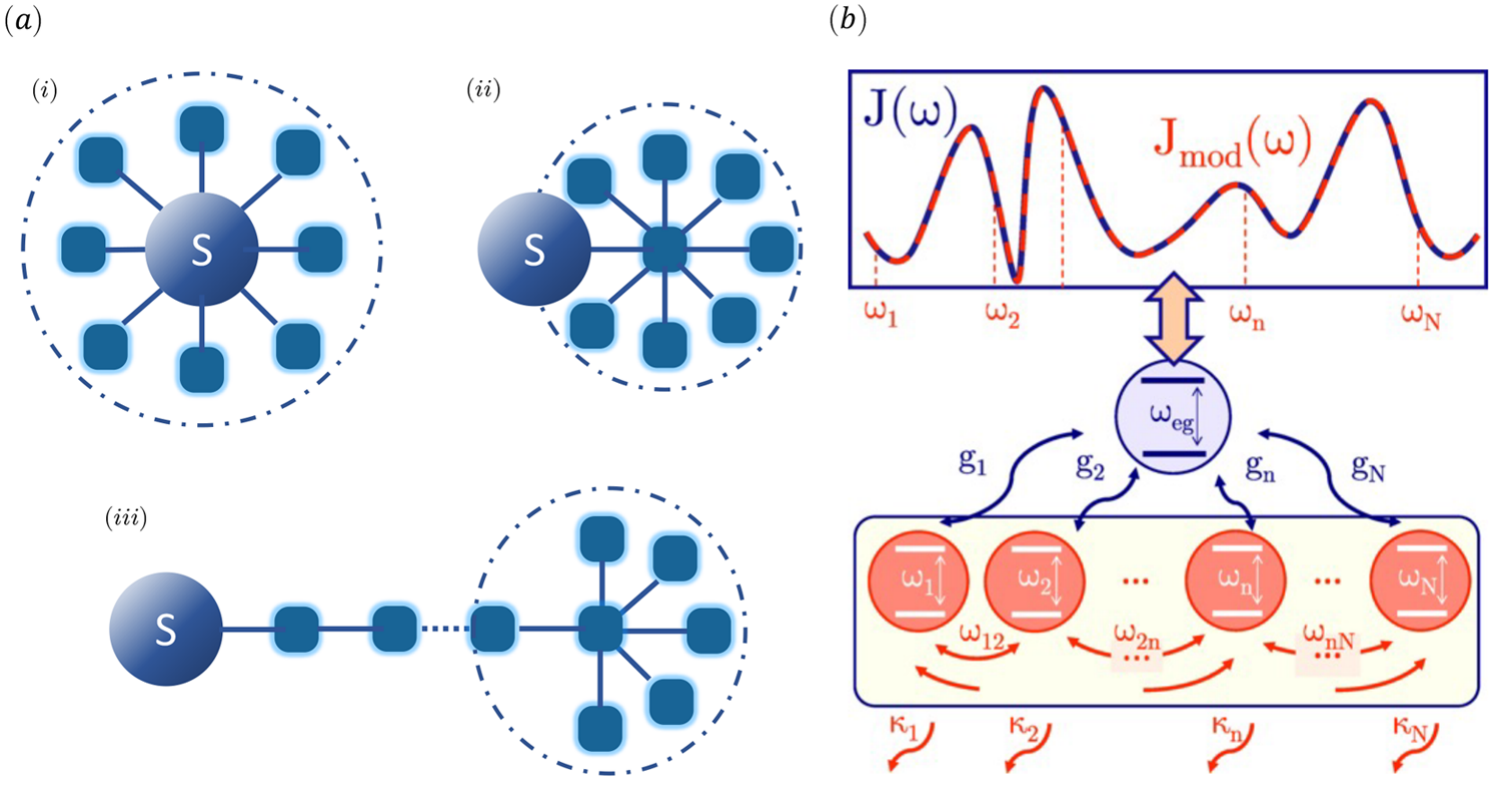
(a) Sketch of the chain mapping model: (i) quantum system coupled to a discrete set of environment modes; (ii) quantum system coupled to the (collective) reaction mode, with this mode coupled to a residual bath of modes; (iii) chain mapping for the environment modes after *n* steps, with a residual bath of *N* – *n* modes at the end of the chain. (b) Sketch of the few-mode quantization model for one quantum emitter. The spectral density of the model can be fitted to the one obtained classically from the Green’s function. Therefore, the couplings to the interacting modes *g*_*i*_, their frequencies and coupling ω_*ij*_, and the dissipative terms κ_*i*_ can be known. Panel (a) is reprinted from ref (98) under the terms of the Creative Commons Attribution 4.0 International license. Copyright 2021 MDPI. Panel (b) is reprinted from ref (99) under the terms of the Creative Commons Attribution 4.0 International license. Copyright 2021 American Physical Society.

For a single emitter with only a single relevant dipole transition (i.e., a two-level system), the matrix-valued generalized spectral density reduces to a scalar function. It is then customary to include the (single) dipole transition matrix element into the definition, giving the conventional scalar spectral density
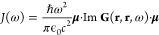
7where **μ** is the transition dipole moment and **r** is the position of the single emitter. A schematic picture of this physical magnitude is rendered in the top panel of Figure 3b. For the many situations where the classical EM spectral density can be used, i.e., in systems where the electronic wave function overlap between material elements and emitters is negligible, the EM environment is a continuum that can be treated as a bath. This makes all the theoretical tools of the field of open quantum systems available. As noted above, in the weak-coupling regime, the bath can be treated perturbatively through the Markov approximation, which just induces level shifts (often assumed to be included in the emitter frequency ω_e_ and thus neglected) and radiative decay with rate γ_r_ = 2*πJ*(ω_e_).

In the more interesting situation where the Markovian approximation is not applicable, there are several available methods to treat a bath of harmonic oscillators (the photon modes) exactly using advanced computational tools. One possibility is to solve the Heisenberg equations of motion while adding stochastic quantum noise.^100^ Another alternative is the cumulant expansion,^101−103^ which is based on solving the Heisenberg equations of motion for the correlations between operators and truncating the resulting expansion at a given order. It is in this sense an extension of mean-field approaches to arbitrary order. For small numbers of molecules (or a single one), relatively high orders of the correlations are needed to accurately describe the dynamics even in the presence of photonic continua,^104^ while for large numbers of molecules, the expansion converges much earlier.^105^ For cases where it works, this expansion provides a relatively low-cost approach that can handle situations that are difficult to treat using other approaches. For example, it can predict the effects of fluctuations on the lasing behavior of an organic-molecule polaritonic system taking into account the multimode character of the cavity, which leads to a switching of lasing behavior between different polaritonic modes, either those resonant with maximal gain or those at the bottom of the polariton dispersion.^105^

Several other commonly used approaches rely on the so-called chain mapping, an orthogonal transformation that maps the Hamiltonian of one emitter coupled to a continuum of modes to a chain-like Hamiltonian where the emitter is only coupled to the first site, which corresponds to a reaction mode, i.e., a collective environment mode, in an infinite string of modes coupled through nearest-neighbor interactions.^106−108^Figure 3a shows a sketch of this transformation. In this form, tensor network approaches that represent a high-dimensional wave function as a product of many lower-dimensional matrices (a so-called matrix product state) become highly efficient as the entanglement in an effective 1D system such as a chain is limited. Tensor network approaches depend exactly on a truncation of the possible entanglement between different parts of the system. In the context of molecular polaritonics, they have been shown to allow the description of several molecules coupled to complex environments.^109−111^ A fully converged tensor network calculation gives exact results, but becomes computationally challenging when long propagation times are desired as the entanglement grows over time. Furthermore, the formally infinite chain has to be truncated at finite length in any realistic calculation, with the required length increasing with propagation time (to prevent unphysical reflections from the end of the chain). In order to decrease the length of the chain and access long times with low computational effort, it is possible to introduce fictitious losses along it that lead to damping of the propagating excitations (similar to the absorbing potentials used in many areas of physics).^98^ Another approach is to employ transfer tensors, which can be used to propagate to arbitrary times with linear computational cost,^112^ or the construction of process tensors and their recompression through tensor network techniques.^113^

While the description of the EM modes as a structured continuum described by the spectral density is formally exact, it is often advantageous and desired to obtain a description of the environment in terms of a few discrete modes, corresponding to the physical image of isolated cavity modes. When losses are included, these are not true eigenmodes of the system, but resonances with a given line width embedded in the continuum. Several methods to achieve such a few-mode quantized description have been developed in the past few years. One is based on quasinormal modes, which are eigenmodes of the Maxwell equations including losses with complex frequencies.^114,115^ They can be used to expand the electric field solutions based on a master equation approach^116^ or explicitly quantized such that the EM field is represented in terms of discrete bosonic modes.^117,118^ In this approach, the discrete modes are defined as superpositions of the bosonic field operators **f̂**_λ_(**r**, ω) of macroscopic QED with coefficients determined by the quasinormal modes obtained from classical EM calculations, and the resulting modes are orthonormalized to obtain approximate discrete lossy modes.

An alternative approach that does not require calculation and explicit quantization of quasinormal modes is based on the fact that two systems with the same spectral density are indistinguishable for an emitter. This allows the construction of a model system consisting of a few coupled discrete modes that are themselves coupled to a background bath and reproduce the full spectral density.^99^Figure 3b shows a sketch of that model. The parameters of the model, which are obtained through fitting of the spectral density, are the coupling between the emitter and the discrete modes *g*_*i*_, the frequencies of these modes and their couplings ω_*ij*_, and their dissipation κ_*i*_. The coupling to the background modes is spectrally flat (by construction) and thus leads to Markovian dynamics that can be represented in a Lindblad master equation, such that the full EM continuum is represented by a collection of lossy and coupled discrete modes in the master equation. This approach is not only computationally efficient but also allows describing the EM environment in the language of cavity QED and quantum optics. The fact that the discrete modes are mutually coupled makes it able to reproduce even complex interference phenomena between the EM modes in the spectral density. This is especially relevant for hybrid cavities where different types of EM modes (such as localized plasmonic resonances and standing-wave modes) interfere. The coexistence of several interacting and decaying modes in such subwavelength cavity QED systems can lead to complex emitter and field dynamics that are not captured by standard models of quantum optics and cavity QED and are only starting to be explored. While originally developed for a single emitter,^99^ we have recently extended the approach to the case of several emitters^96^ or one emitter with several contributing transitions (such as different orientations of the dipole moments). A similar model as in the single-emitter case can then be used to fit the generalized spectral density, eq 6. This approach is then able to capture the effect of a complex mode structure on emitter dynamics and, for example, population transfer between emitters. As an example, it can describe how the dynamics of population transfer between two emitters coupled through a multimode cavity can be modified by the presence and excitation state of a third emitter.^96^

## 3 Introducing Molecular Complexity

For the description of the molecules, we focus on approaches that are well-adapted for describing “good” molecular emitters, meaning ones where the first electronically excited state is relatively stable against nonradiative decay and photochemical reactions do not take place. For such emitters, it is often a good approximation to represent the nuclear potential energy surfaces as harmonic oscillators, which significantly simplifies the treatment. When more chemical detail is needed, a wide variety of methods are nowadays available, but doing so typically limits the level of description of nuclear motion. A discussion of such methods focusing on chemically accurate descriptions can be found in a related recent perspective on polaritonic chemistry.^8^

As detailed in the Introduction, the simplest approach is to treat molecules as two-level systems. This can be well-justified for studies at cryogenic temperatures where vibrational sublevels are individually resolved and addressable.^49,119^ At room temperature, the influence of the vibrational motion of the molecule can be approximately included by adding a pure dephasing term in a Lindblad master equation description. This can be understood as arising from an exciton–vibration coupling term treated through the Markov approximation. Such a treatment allows modeling the influence of molecular vibrations on processes where vibration-induced transitions between the polaritons and dark states do not dominate the dynamics. One such example is coherent polariton-mediated long-range exciton conductance (i.e., energy transport).^120,121^ In such systems, it was found that the coherent nature of the polaritons only becomes realized and significantly enhances transport when the Rabi splitting significantly surpasses the dephasing rate.

While modeling pure dephasing through a Lindblad term can give some insight into the vibration-induced decoherence of the molecules, this treatment is not fully correct. Since under strong coupling the molecular exciton state gets distributed over the polaritonic modes, the Markov approximation that was originally performed for the emitter by itself to obtain a pure dephasing term is not valid anymore.^122^ Including it without further modification in the strongly coupled system leads to artificial population transfer between the polaritons and dark states, with equal rates for pumping of energy from the reservoir of vibrations to the system as for loss of energy from the system to the reservoir. This is unphysical when the Rabi splitting is comparable to or larger than the thermal energy, a condition that is essentially always fulfilled in molecular exciton–polariton strong coupling. This problem can be resolved by applying the Markov approximation after taking into account the strong coupling, e.g., by using a Bloch–Redfield approach.^79,80,123,124^ This approach captures the fact that vibration-induced transitions between states preferably lead to relaxation, i.e., loss of energy to the bath of molecular vibrations. This one-way transfer to the lowest state in the excitation manifold can be exploited to selectively excite states with a desired characteristic in polaritonic systems and in particular can be exploited to induce long-range energy transfer between different molecular species driven by their local vibrations and enabled by the nonlocal character of the polaritons.^5,6,78−82^

When the exciton–phonon coupling is sufficiently strong that the above approach breaks down, it becomes necessary to explicitly include the vibrational modes of the molecules. The simplest approach is the so-called Holstein model, which treats only a single vibrational mode and describes each molecule as two displaced harmonic oscillators. For multiple emitters, this leads to the so-called Holstein–Tavis–Cummings model,^125,126^ within which each electronic level is represented by several vibrational sublevels. Within this model, the effect of the vibronic coupling can be studied, so an analysis of nuclear dynamics in molecules under strong coupling is feasible. Along this line, it has been predicted that electron transfer between different excited states can be enhanced or suppressed.^126^ The physical effect behind these changes is a decoupling of vibrational modes from the polaritons due to the collective nature of the excitation. Similar effects also occur in molecular J- and H-aggregates outside of cavities, where the collective nature arises due to dipole–dipole interactions between the monomers and leads to significantly reduced line widths.^127^ Within the Holstein–Tavis–Cummings model, the inclusion of vibronic sublevels and the concomitant emergence of dark vibronic polaritons (collective light–matter states that weakly absorb but strongly emit radiation) allows for a better description of the spectroscopy and dynamics of organic microcavities in the strong-coupling regime.^128−130^ The inclusion of vibrational levels is also highly relevant for the description of phenomena such as organic polariton lasing and polariton condensation.^125,131,132^

**Figure 4:**
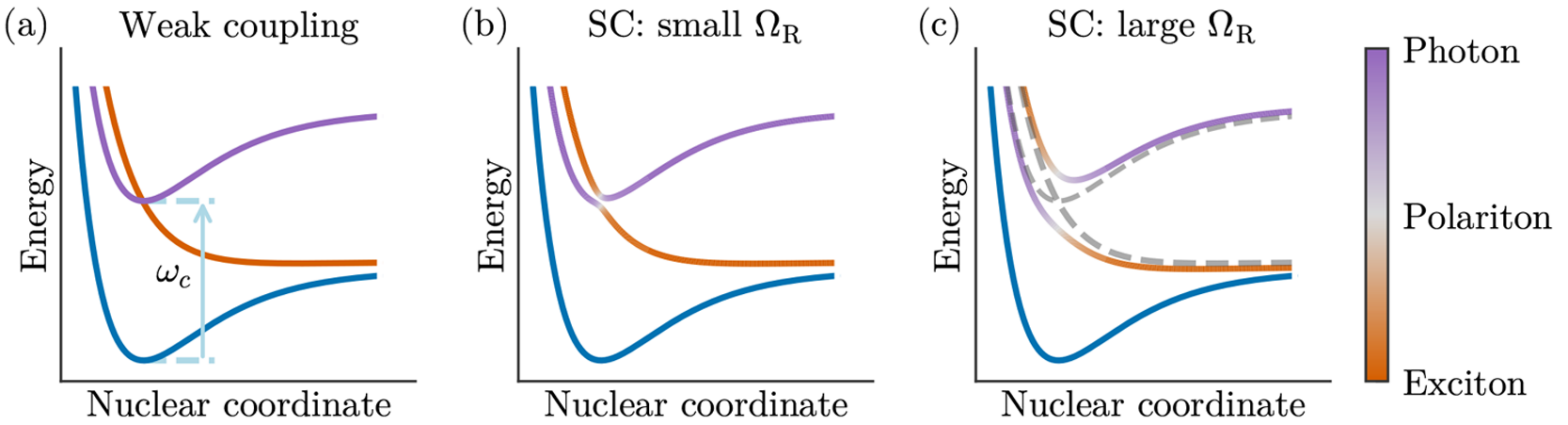
Conceptual potential energy surfaces for one cavity mode and one molecule at different coupling strengths: (a) weak coupling; (b, c) strong coupling. The color represents the fraction of each constituent. Reprinted with permission from ref (51). Copyright 2017 American Chemical Society.

An extension of the Holstein–Tavis–Cummings model that allows for more realistic molecular structure is to consider more complex potential energy curves instead of harmonic oscillators, permitting the treatment of vibrational nonlinearities and (photo)chemical reactions within a relatively simple model (especially if only a single vibrational degree of freedom is treated). The hybridization of the potential energy surfaces in the strong coupling regime then leads to hybridized polaritonic potential energy surfaces (PoPES), which have mixed photon–matter properties.^51,133,134^ A schematic representation of these PoPES can be found in Figure 4. Within this approach, the polariton decoupling of the vibrational modes can be easily understood from the fact that a single excitation is distributed over many molecules in a polaritonic state; that is, each molecule is only excited with a small probability amplitude and spends most of the time in its electronic ground state. The polaritonic potential energy surface then follows the ground-state one. This fact allows designing new potential energy landscapes in the coupled system by “copying and pasting” together excited-state and ground-state-like potentials from different nuclear configurations depending on whether the system is on resonance with the cavity or not at the specific configuration.^134^

Assuming that vibrational modes are harmonic oscillators but taking into account all degrees of freedom for the molecules (typically hundreds), and potentially of the surrounding solvent or polymer host material, one can again rely on tensor network techniques as discussed above for the photonic modes. The molecular vibrations are then represented by an independent chain of harmonic oscillators.^109−111,135^ This approach describes the initial coherent oscillation of the nuclear wavepacket and its influence on the polariton dynamics, as well as its gradual suppression due to the presence of a multitude of modes. When the exact dynamics of the vibrational modes are not desired, the bath of harmonic vibrational modes can be represented through its correlation function and simulated using the time-evolving matrix product operator (TEMPO) method.^136,137^ This uses a tensor network to describe the system’s history over a finite memory time and can thus represent non-Markovian dynamics. Combining this technique with a mean-field approximation further reduces the problem size.^138^ This method can thus describe the vibration-induced dynamics and dephasing of the molecular wavepackets over long times within a fully quantized approach.

We note at this point that although many effects can be understood by the use of the previous models, all of them constitute strong approximations for the molecular structure. In particular, the use of harmonic oscillators to describe the vibrational modes precludes the description of any nonlinear vibrational effects or of chemical transition states, conical intersections, etc. However, a full quantum description of the molecules is an extremely challenging task and only possible for small molecules. To give a more complete picture by including all degrees of freedom inside the molecules requires utilizing quantum chemistry and ab initio approaches.^8^

## 4 Summary and Outlook

In this article we have provided a perspective on the current status of the theoretical investigation devoted to analyzing the exciting physics in the emergent field of molecular polaritonics. This area of research deals with the strong light–matter coupling regime that appears between electronic/vibrational excitations within (organic) molecules and confined light fields. As for the light field component, a photonic structure that acts as a cavity is needed. Depending on the cavity used, strong coupling can be reached by utilizing a large ensemble of molecules or just one or a few of them, depending on whether or not subwavelength confinement is achieved.

The theoretical description of the EM modes that arise in these photonic structures, which in general are lossy, is then a challenging task. When the cavities present small losses, they can be treated perturbatively, while for small enough subwavelength cavities, the quasistatic approximation can be used, which allows for semianalytical solutions. However, in many physical situations, a fully quantized description of the EM field in photonic structures is required. Here we have shown how the macroscopic QED formalism provides the necessary theoretical and numerical tools to accurately describe light–matter strong coupling in arbitrary structures. Within this framework, the spectral densities that characterize the coupling between one or several quantum emitters and confined EM modes are fully determined by the classical Green’s functions, which are calculated using standard numerical solvers of Maxwell’s equations in complex EM media. Based on macroscopic QED, it is then feasible to develop approaches that allow for a tractable treatment of complex photonic environments. Among these simplified treatments, the most promising approaches are those based on the concept of quasinormal EM modes and a very recent one that relies on the construction of a model system involving a small number of lossy and interacting EM modes whose parameters are fitted to exactly reproduce the spectral densities associated with the photonic structure under study.

Regarding the matter component, organic molecules also have a complex internal structure, which prevents them from being theoretically modeled as just two-level systems in many situations. To include molecular complexity in the theoretical description, it is then mandatory to add some ingredients to the standard two-level model to describe the vibrational modes of the molecules. Depending on the strength of the vibronic coupling, a pure dephasing term, the Bloch–Redfield approximation, or the explicit inclusion of the vibrational modes needs to be utilized. Within this last approach, the most used framework is the so-called Holstein–Tavis–Cummings model, which only takes into account one vibrational mode, modeled as a harmonic oscillator, which has proven to be very successful in providing physical insight. Going beyond this model can be achieved either by including more of the (typically hundreds of) vibrational modes of a molecule or by substituting the harmonic oscillators by more realistic potential energy surfaces. The first approach captures vibration-induced dephasing and decoherence, while the second naturally accounts for the vibrational nonlinearities and has also allowed for a fundamental description of (photo)chemical reactions induced by strong light–matter coupling. Nevertheless, in order to have a more accurate description of the internal structure of organic molecules, numerical formalisms that rely on quantum chemistry codes or ab initio approaches need to be utilized.

At this stage, for the case of collective strong coupling in which a large (macroscopic) number of molecules is involved, the current status of the theoretical research on molecular polaritonics does not allow for both a realistic treatment of the internal structure of the organic molecules and a detailed account of the complex EM media. This is indeed very frustrating, as the majority of the experiments carried out in this field belong to the category of collective strong coupling. Therefore, a quantitative agreement between ab initio theory and experiment is not within reach nowadays. As a concrete example, current ab initio theoretical models do not correctly predict the experimentally observed decay rates from the exciton reservoir to the lower polariton. This is why during the past decade most theoretical research has focused either on giving fundamental support to some of the experimental findings or to propose new effects that result from theoretical approaches based on simplified models. On a more positive note, for the case in which only a single or few molecules participate in the strong-coupling phenomenon, after 10 years of intense research, we now have the adequate theoretical and numerical tools to accurately describe both the internal vibrational modes of the organic molecules and the complexity of the subwavelength EM fields associated with nanophotonic (mainly plasmonic) structures. We expect that these theoretical developments will help to open new and exciting avenues for research in molecular polaritonics.
